# Engineering Improves Enzymatic Synthesis of L-Tryptophan by Tryptophan Synthase from *Escherichia coli*

**DOI:** 10.3390/microorganisms8040519

**Published:** 2020-04-05

**Authors:** Lisheng Xu, Fangkai Han, Zeng Dong, Zhaojun Wei

**Affiliations:** 1 Department of Life and Food Science, Suzhou University, Suzhou 234000, China; hanfk11@ahszu.edu.cn (F.H.); dongzeng@ahszu.edu.cn (Z.D.); 2 School of Food and Biological Engineering, Hefei University of Technology, Hefei 230009, China; zjwei@hfut.edu.cn

**Keywords:** tryptophan synthase, thermostability, L-tryptophan, directed evolution

## Abstract

To improve the thermostability of tryptophan synthase, the molecular modification of tryptophan synthase was carried out by rational molecular engineering. First, B-FITTER software was used to analyze the temperature factor (B-factor) of each amino acid residue in the crystal structure of tryptophan synthase. A key amino acid residue, G395, which adversely affected the thermal stability of the enzyme, was identified, and then, a mutant library was constructed by site-specific saturation mutation. A mutant (G395S) enzyme with significantly improved thermal stability was screened from the saturated mutant library. Error-prone PCR was used to conduct a directed evolution of the mutant enzyme (G395S). Compared with the parent, the mutant enzyme (G395S /A191T) had a *K*_m_ of 0.21 mM and a catalytic efficiency *k*_cat_/*K*_m_ of 5.38 mM^−1^∙s^−1^, which was 4.8 times higher than that of the wild-type strain. The conditions for L-tryptophan synthesis by the mutated enzyme were a L-serine concentration of 50 mmol/L, a reaction temperature of 40 °C, pH of 8, a reaction time of 12 h, and an L-tryptophan yield of 81%. The thermal stability of the enzyme can be improved by using an appropriate rational design strategy to modify the correct site. The catalytic activity of tryptophan synthase was increased by directed evolution.

## 1. Introduction 

Tryptophan is a precursor of serotonin, an important neurotransmitter, and an essential amino acid in the human body. Tryptophan synthetase (EC 4.2.1.20) is a heterotetramer with an aaββ subunit structure in *Escherichia coli*. The enzyme can synthesize L-tryptophan with indole and L-serine as substrates. In L-tryptophan metabolism, tryptophan synthase is one of the important enzymes that affect the yield of L-tryptophan. The *trp B* and *trp A* genes (or *trp BA* genes) coexist in the tryptophan operon of the *E. coli* genome. The function of the a subunit is to decompose indole-3-glycerol phosphate into indole and glyceraldehyde-3-phosphate, while the function of the β subunit is to synthesize L-tryptophan [1,2,3]. The substrate spectra of tryptophan synthase derived from *Salmonella*, including fluorine, chlorine, bromine, and iodine-substituted indole derivatives, have been reported in the literature. This enzyme can synthesize L-tryptophan derivatives [4]. The active sites of tryptophan synthase in *Pyrococcus* *furiosus* mainly include Asp300, Glu104, Lys82, and Ala106, among which hydrogen bond formation between substrate complex E(Aex1) and Asp300 plays an important role in the catalytic process. The tryptophan synthase from *Pyrococcus furiosus* was modified via directed evolution, and the ability of the enzyme to synthesize 5-bromo-L-tryptophan and 6-hydroxy-L-tryptophan was promoted by modification of the tryptophan synthase β subunit [5]. S-phenyl-L-cysteine and L-2-methyltryptophan were synthesized by tryptophan synthase [6,7,8]. However, modification of the tryptophan synthase derived from *E. coli* K-12 has not been reported. In recent years, the molecular modification was applied to enzyme catalysis [9,10,11,12]. Useful compounds such as 5-aminolevulinic acid, ginsenoside, and L-theanine can be synthesized from microorganisms [13,14,15]. Frances Arnold proposed the concept of directed evolution of enzymes in the 1990s. Directed evolution greatly broadens the biocatalytic potential of an enzyme by modifying its corresponding gene sequence, enabling it to possess a wide range of substrate universality and even catalyze entirely new chemical reactions. Through the directed evolution of the cytochrome P411 enzyme, dicyclobutane, and cyclopropylene were efficiently synthesized [16]. The thermal stability, spatial selectivity, and activity of epoxide hydrolase were optimized by iterative saturation mutation and triplet codon saturation mutation strategies [17]. An efficient protein engineering strategy based on the fusion of directed evolution and rational design was used to obtain lipase mutant strains with high stereoselectivity, and the strategy required minimal screening. Crystal structure analysis and molecular dynamics simulation of the nonaqueous phase were used to explain the specific stereoselectivity and the molecular mechanism of the four mutant strains, respectively [18]. Directed evolution was used to modify threonine aldolase to improve the synergistic effect of its amino acid residues [19]. High stereoselective mutations in the R and S isomers of a-2-aryl propionate were obtained by iterative saturation mutation directed evolution of its active sites. Nearly 1000 mutant strains of *Candida antarctica* lipase B were selected for high-throughput screening [20]. By mutating transaminase, its biocatalytic activity increased by 25,000 times, and the modified transaminase could be used in the green production of the hypoglycemic drug citastatin [21]. The biosynthesis pathway of tryptophan in *Escherichia coli* mainly includes the central metabolic pathway, a common pathway, and a branch pathway. In the central metabolic pathway, the *ppsA* and *tktA* genes play key roles in the generation of phosphoenolpyruvate, precursors of tryptophan biosynthesis. In the common pathway, 3-deoxy-D-arabino-heptulosonate-7-phosphate (DAHP) synthase catalyzes the condensation of E4P and phoshoenolpyruvate to produce DAHP as a rate-limiting step. In the branching pathway, anthranilic acid synthetase is the key enzyme in tryptophan biosynthesis [22]. The molecular kinetics of the catalysis of tryptophan synthetase have been studied. The structure and reaction mechanism of tryptophan synthetase from Salmonella typhimurium have been analysed by NMR [23,24]. Excellent thermostability and catalytic activity of tryptophan synthase were needed in industrial production. In this study, tryptophan synthase derived from *Escherichia coli* K-12 was modified by directed evolution and rational design to improve its activity and thermostability. L-tryptophan was synthesized by modified tryptophan synthetase.

## 2. Materials and Methods

### 2.1. Chemicals, Strains and Culture Conditions

The plasmid extraction kit, DNA polymerase, indole, and isopropyl-beta-D-thiogalactopyranoside (IPTG) were purchased from Sangon Biotech (Shanghai, China) Co., Ltd. *E. coli* BL21(DE3) and the expression plasmid pet-28a (+) were preserved in our laboratory.

### 2.2. Mutation Site

Reetz et al. successfully improved the stability of *Bacillus subtilis* lipase based on B-FITTER [18]. In this study, B-FITTER software was used to analyze the temperature factors of 397 amino acid residues of tryptophan synthase trpB (PDB: 2dh6), and the temperature factors were obtained. The temperature factors of the amino acid residues within the entrance range of the substrate channel were analyzed by a Swiss-model online simulation of the three-dimensional structure. According to the temperature factor, the key amino acid residues related to substrate binding were screened out.

### 2.3. Site-Saturated Mutation Library

pET-28a (+) was used as the template plasmid for site-specific saturation mutation of 395 amino acids. The primers used for mutagenesis are presented in Table 1. The PCR reaction system included 10× PCR buffer solution, 2.5 mmol·L^−1^ dNTP mixture, 15 pmol·L^−1^ primers, pET28a(+)-trp B, and 5 U·L^−1^ DNA polymerase (PCR reaction system 50 μL). The PCR reaction conditions included denaturation at 95 °C for 4 min, followed by 20 cycles of 95 °C for 30 s, 55 °C for 1 min, and 72 °C for 16 min, and finally, 72 °C for 5 min. The PCR products were enzymatically digested at 37 °C for 4 h to eliminate the parent template, and the enzymatic hydrolysis products were transformed into *E. coli* BL21(DE3) competent cells by the calcium chloride method. The transformation liquid was coated with agar medium containing 50 μg·L^−1^ kanamycin to obtain the site-saturated mutant library.

### 2.4. Error-Prone PCR Mutant Library

With pET-28a (+)-trpB as the template, the trans-EasyTaq DNA Polymerase kit was used to introduce random mutations in the full-length trpB gene. Recombinant mutant plasmids were constructed. The genes were synthesized by Sangon Biotech (Shanghai, China) Co., Ltd. The target gene was constructed between the BamH I and Hind III cleavage sites of the pET-28a (+) vector. Successfully transformed clones were inoculated in 5 mL LB liquid medium (50 μg·mL^−1^ Kan), were cultured at 37 °C and 200 r·min^−1^ for 8 h and were then transferred to 50 mL LB liquid medium (50 μg·mL^−1^ Kan). IPTG was added at 18 °C for induction. The mutant library was obtained by transforming *E. coli* competent cells.

### 2.5. Screening of Mutated Enzymes

LB liquid medium (200 mL) was added to each well of the 96-well plate (50 μg·mL^−1^ Kan). A single colony was selected from the plate with a sterilized toothpick and inoculated into the 96 shallow orifice plate (37 °C, 200 r·min^−1^, 3 h). IPTG was added at 18 °C for induction. The fermentation broth was centrifuged (4 °C, 4000 r·min^−^^1^,10 min). The cells were washed with PBS buffer and were then added to the reaction plate according to the corresponding position. L-Serine (10 mmol·L^−^^1^) was added to the reaction plate (37 °C, 30 min, 200 r·min^−^^1^). After the reaction, the reactant was centrifuged (4 °C, 4000 r·min^−^^1^,10 min). The absorbance value at 290 nm was determined by ELIASA, and the mutant strain with an absorbance value that increased relative to the absorbance value of TrpB was selected for re-screening.

### 2.6. Protein Purification

To purify tryptophan synthase, the supernatant was applied to a Ni-NTA column based on the methods described in the literature [25]. The quality of purified tryptophan synthase was analyzed by SDS-PAGE.

### 2.7. Synthesis and Identification of L-tryptophan

L-tryptophan (Alighting reagent Co., Ltd., Shang hai, China) was synthesized by tryptophan synthase from L-serine and indole (50 mmol/L L-serine, 50 mmol/L indole, 40 °C, pH 8, 12 h). L-tryptophan was analyzed with an FT-IR Spectrometer ( Gangdong Sci & Tech. Co., Ltd., Tianjing, China), Nuclear Magnetic Resonance Spectrometer (Bruker Avance 500 MHz, Switzerland), and Liquid Chromatography-Mass Spectrometer (Agilent, Palo alto, California, USA). SPSS 22.0 (SPSS, Chicago, Illinois, USA) was used to calculate the standard error and the significance of differences.

## 3. Results

### 3.1. Selection of Mutation Sites

B-FITTER software was used to analyze 397 amino acid residue sites of tryptophan synthase and obtain their temperature factor values. Then, the temperature factor values of the amino acids at the entrance of the substrate channel were sorted, and the top 20 amino acid residue sites were identified (Table 2). Among these 20 amino acid residues, we selected glycine 395 (G395), as it was the site with the largest temperature factor value for molecular modification.

### 3.2. Mutation Library Screening

A site-saturated mutant library consisting of 300 mutants was constructed, and a mutant enzyme with significantly improved thermal stability was obtained by initial screening and rescreening. DNA sequencing revealed that the amino acid residue at position 395 of the mutant enzyme was mutated from glycine to serine (the codon was mutated from GGT to AGT).

### 3.3. Optimum Temperature of the Mutant Enzyme

**Figure 1:**
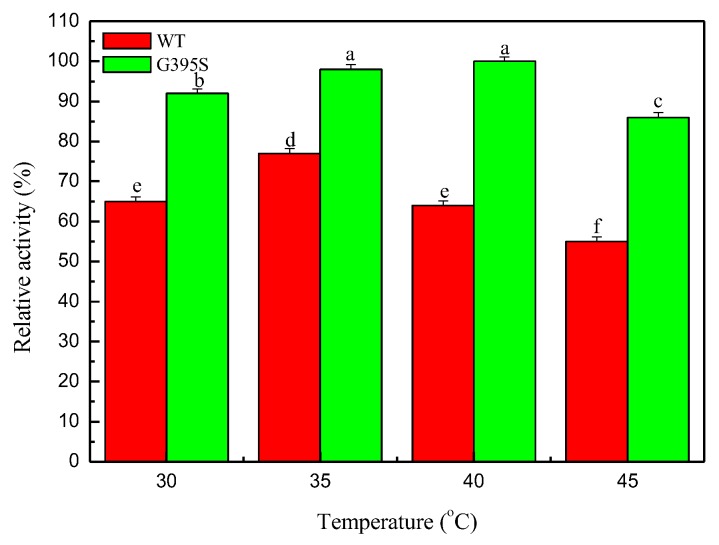
Effects of temperature on the activities of the tryptophan synthase mutant and wild type. Relative activity of the tryptophan synthase mutant (G395S) and wild type (WT) catalyzing reactions at different temperatures (30, 35, 40, and 45 °C), L-serine concentration of 50 mmol/L, pH of 8, 12 h. The Error bars were mean values of three independent replicate. The meaning of letters a, b, c, d, e, f, in the figure legend indicates significant differences.

In the same reaction system, the activities of the mutant enzyme and the parent enzyme at different temperatures (the absorbance increment caused by the formation of the product L-tryptophan was taken as the indicator) were investigated. The results showed that the optimal reaction temperature of the parent enzyme was 35 °C, while the optimal reaction temperature of the mutant enzyme was approximately 40 °C, which was 5 °C higher than that of the parent enzyme (Figure 1). The increase in the optimum reaction temperature indicated that the thermal stability of the mutant enzyme G395S was better than that of the parent enzyme.

### 3.4. Directed Evolution

**Figure 2:**
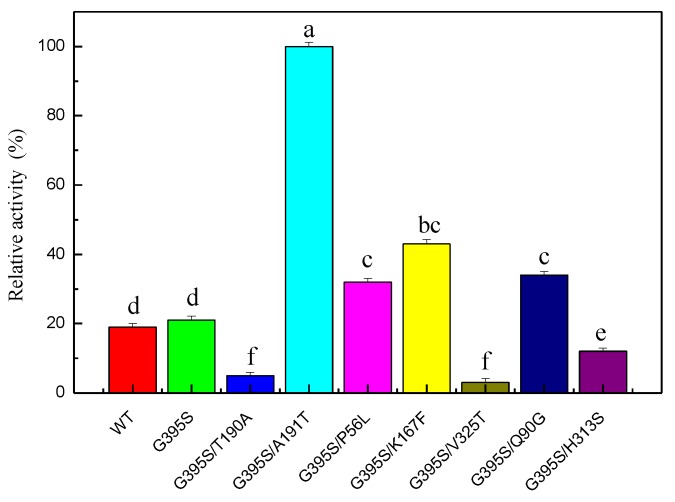
Relative activities of glycine-substituted mutants of tryptophan synthase. The reaction conditions for the mutated enzyme were an L-serine concentration of 50 mmol/L, a reaction temperature of 40 °C, pH of 8, 12 h. The Error bars were mean values of three independent replicate. The meaning of letters a, b, c, d, e, f, in the figure legend indicates significant differences.

Error-prone PCR was used to construct a random mutant library. After screening 15,000 mutant strains, the positive mutant strain G395S/A191T was obtained. The enzyme activity of the highly active positive mutant G395S/A191T was 4.8 times higher than that of the parent enzyme, indicating that sites 191 and 395 were the key sites affecting the enzyme activity of TrpB (Figure 2). The OD600 values for the growth of the wild type and G395S and G395S/A191T mutants were measured. There was no difference in OD600 value during the growth of the three strains, and no differences in the morphology of the three strains were observed under a microscope. Compared with the parent, the mutant enzyme (G395S /A191T) had a *K*_m_ of 0.21 mM and a catalytic efficiency *k*_cat_/*K*_m_ of 5.38 mM^−1^∙s^−1^, which was 4.8 times higher than that of the parent (Table 3).

### 3.5. Synthesis of L-tryptophan

**Figure 3:**
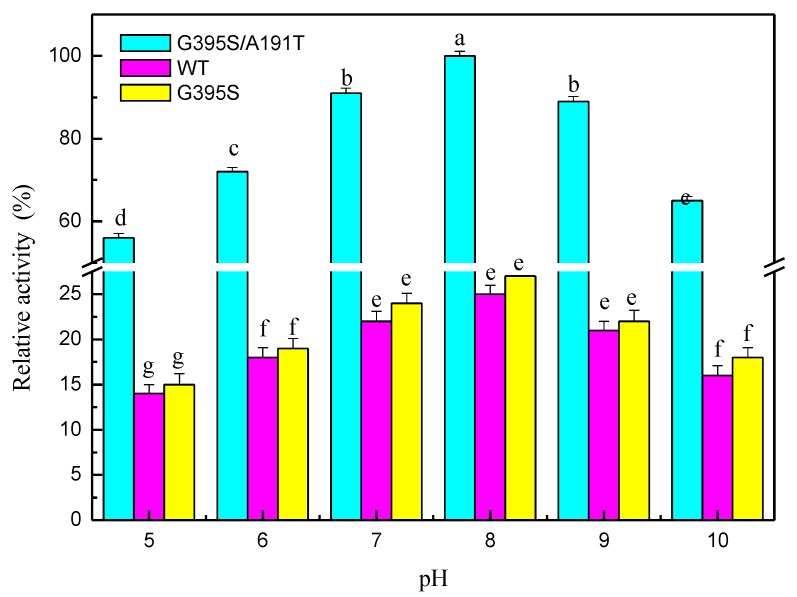
Effects of pH on tryptophan synthase activity. pH stability of tryptophan synthase at different pH values (5, 6, 7, 8, 9, 10), L-serine concentration of 50 mmol/L, 40 °C, 12 h. The Error bars were mean values of three independent replicate. The meaning of letters a, b, c, d, e, f, g, in the figure legend indicates significant differences.

**Figure 4:**
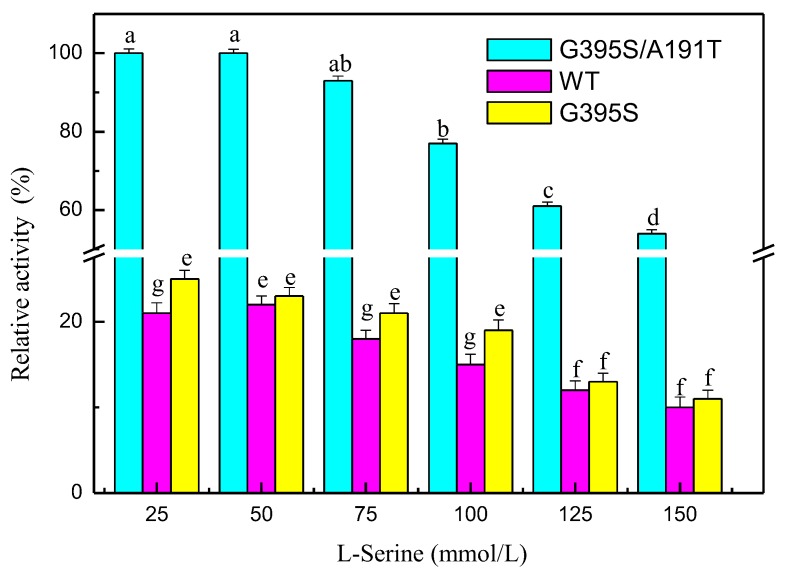
Effects of the L-serine concentration on tryptophan synthase activity. Relative activity of tryptophan synthase at different L-serine concentrations (25, 50, 75, 100, 125, 150 mmol/L), 40 °C, pH of 8, 12 h. The Error bars were mean values of three independent replicate. The meaning of letters a,b,c,d,e,f,g, in the figure legend indicates significant differences.

**Figure 5:**
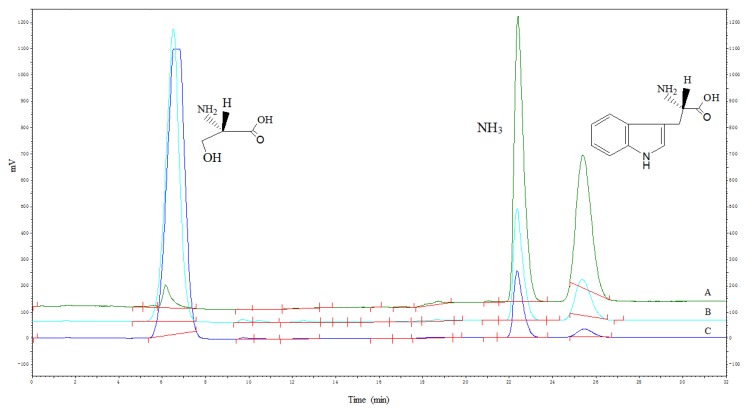
Synthesis of L-tryptophan by the three strains. The reaction conditions for three tryptophan synthase recombinant strains ((**A**): G395S /A191T; (**B**): G395S; (**C**): Wild strain) were an L-serine concentration of 50 mmol/L, reaction temperature of 40 °C, pH of 8, 12 h. Red line was the integral line.

The mutated enzymes reacted at different pH values, and the results showed that the mutant enzyme G395S/A191T had the highest activity at pH 8. The optimal pH value of the mutant enzyme G395S and the parental enzyme was also 8, but the activities of the mutant enzyme G395S and parental enzyme were lower than that of the mutant enzyme G395S/A191T (Figure 3). These results showed that the mutant enzyme G395S/A191T maintained high activity in the L-serine concentration range of 25, 50, and 75 mmol/L, while the optimal L-serine concentration of the mutant enzyme G395S and parent enzyme was approximately 50 mmol/L (Figure 4). Under the reaction conditions of an L-serine concentration of 50 mmol/L, the production of L-tryptophan was determined by an Amino Acid Analyzer (Hitachi L-8900, Japan). Synthesis of L-tryptophan by the mutant enzyme G395S/A191T was higher than that by the mutant enzyme G395S and parental enzyme (Figure 5).

### 3.6. Identification of L-tryptophan

**Figure 6:**
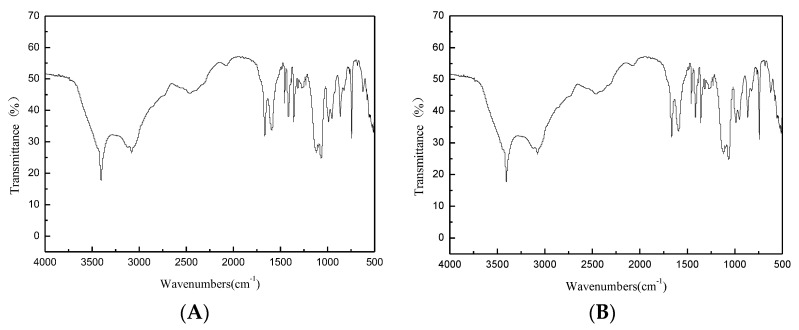
FTIR spectrum of L-tryptophan. ((**A**): Reaction product; (**B**): Standard substance).

**Figure 7:**
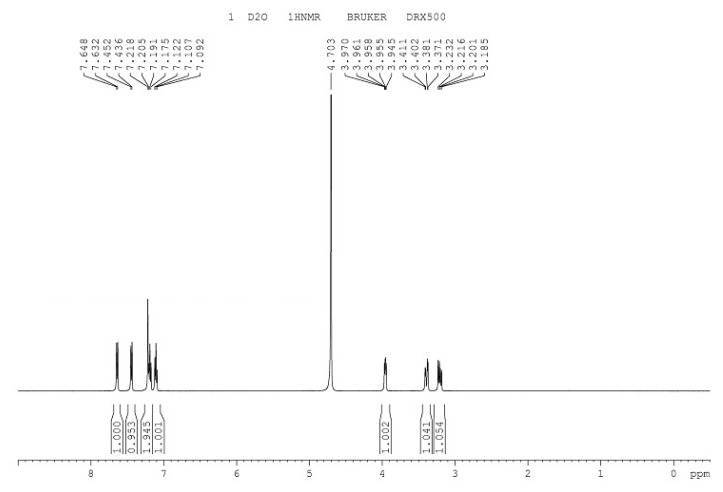
^1^H-NMR spectrum of L-tryptophan.

**Figure 8:**
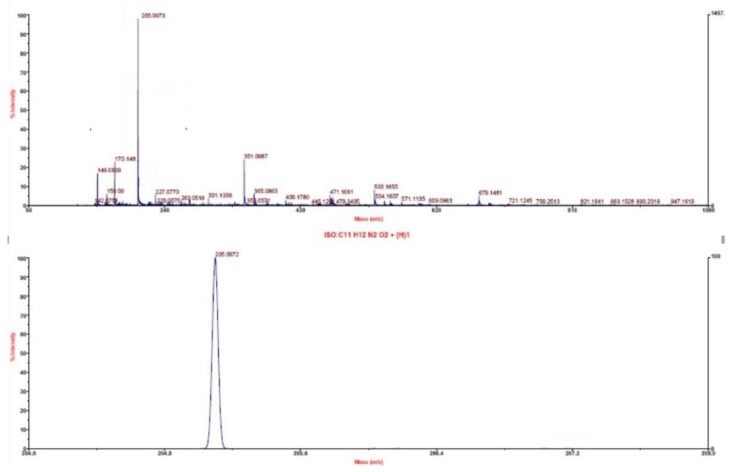
Mass spectrum of L-tryptophan.

The infrared spectrum of L-tryptophan is shown in Figure 6, with the introduction of amino and carboxyl groups. The ^1^H-NMR spectrum of L-tryptophan is shown in Figure 7. ^1^H NMR (500 MHz, D2O): δ 3.23(s, 3H), 3.41 (d, 2H), 3.97 (t, 1H), 7.12 (d, 1H), 7.21 (d,1H), 7.45 (m, H), 7.64 (m, H). The theoretical molecular weight of L-tryptophan is 204 Da. As shown in Figure 8, a signal at m/z 205 appeared in the mass spectrum of the reaction mixture, which is consistent with ionized L-tryptophan (Figure 8).

### 3.7. Mutation Analysis 

**Figure 9:**
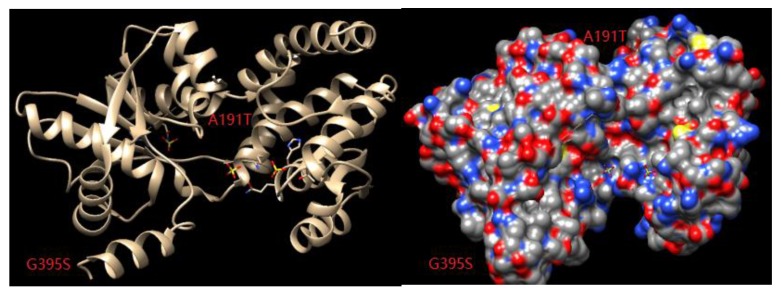
Relationship between the substrate channel and the 191 and 395 binding sites.

Appropriate modifications of the amino acid residues in the substrate channel can promote the recognition and binding abilities of the enzyme. Through a combination of temperature factor analysis and substrate channel localization, key amino acid residues that affect both the thermal stability and catalytic activity of the enzyme can be identified. In combination with saturation site-specific mutation and screening, target mutant enzymes with improved thermal stability and catalytic activity can be obtained from all the possible mutations. Chimera1.13 was used to analyze the substrate channel space of the enzyme, and it was found that the amino acid at position 395 was located in a loop involved in substrate channel formation (Figure 9). After the mutation of this amino acid from glycine to serine, the spatial orientation freedom of the main chain was reduced, the rigidity of the enzyme was increased, and the thermal stability of the enzyme was improved. Alanine at position 191 was mutated to threonine, which may have changed the folding direction of the loop, thus changing the shape and size of the substrate channel entrance and increasing enzyme activity (Figure 9).

## 4. Discussion

According to the structural information of proteins and sequence comparisons of homologous proteins, using a rational selection of amino acid residues as targets combined with site-specific mutation of key amino acids, we can screen and obtain target mutants [26,27,28]. Rational design can improve the substrate specificity of an enzyme by remodeling its active pocket, which is the structural domain of the enzyme responsible for its catalytic function and usually consists of a hydrophobic core. The amino acids in the active pocket can be divided into catalytic amino acids and substrate channel amino acids. Substrate molecules enter the active pocket and interact with the substrate channel amino acids, placing the substrate molecules in a favorable conformation for catalysis and allowing completion of the catalytic reaction under the action of the catalytic amino acids. Changing the substrate specificity of the enzyme can expand the range of enzyme substrates by remodeling the active pocket of the enzyme to form a different conformation with the substrate molecule or by accepting other substrates. The size of the active pocket directly affects the entry and stability of substrate molecules but can also hinder the entry of substrate molecules and prevent the enzyme from accepting more substrates for catalytic reactions. Therefore, the substrate specificity of the enzyme can be modified by regulating the size of its active pocket. For a smaller active pocket, a site-specific mutation can increase the size of the active pocket to accommodate larger substrate molecules, increasing the substrate specificity of the enzyme molecule. The microenvironment of the active pocket, such as its polarity, is critical to the activity of the enzyme. Different environments lead to preferences for different substrates. According to the spatial structure of an enzyme and the properties of its active pocket, it can be rationally designed to allow the target substrate while maintaining its relative activity and stability to better complete the catalytic reaction. By reducing the polarity of the active pocket, the activity of an enzyme against competitive substrates can be reduced, which is beneficial to accumulating target products in metabolic engineering and improving the specificity of enzyme catalysis. By changing the charge state of the active pocket amino acids, the mutated enzyme has a specificity different from that of the wild type. To analyze ethanol dehydrogenase CpRCR, the active pocket was analyzed, and it was found that F285, W286, and W116 constituted the substrate binding pocket of the enzyme. The activity of mutant enzymes was increased by a rational design to increase the size of the substrate-binding pocket [29]. During the study of LfAmDHs, it was found that the two amino residues A113 and T134, located at the distal end of the substrate binding pocket, blocked the opening of the substrate binding pocket, so LfAmDH could only bind smaller substrates. The mutant was obtained by a rational design using site-specific mutations, and the size of the substrate-binding pocket increased the substrate spectrum of the enzyme [30]. Three key regions of lipase CALB were studied to determine its key amino acids: D223 and A281. D223V/A281S was obtained by site-specific saturation mutation, which increased the selectivity of the final product, methyl succinate from 8% to more than 99% [31]. The ketoisopentane decarboxylase of *Lactococcus lactis* was mutated by rational design. When V461 was mutated into serine, the specificity of the downstream ketoic acid was reduced, which was conducive to the production of the precursor material amyl alcohol [32]. Kaempferol was produced by the metabolic engineering of *Saccharomyces cerevisiae* [33]. B-FITTER software was used to analyze the crystal structure of tryptophan synthetase to determine the temperature factor (B-factor) of each amino acid locus and to identify the adverse effects on the thermal stability of the enzyme caused by mutations of the key amino acid G395. Then, at this site, fixed-point saturated mutations were performed to build a mutant library, and from the saturated mutant library, screening mutation was found to result in better thermal stability of the enzyme (G395S). The reaction temperature of the mutant enzyme G395S was 5 °C higher than that of the parent enzyme. Error-prone PCR was used to conduct a directed evolution of the mutant enzyme G395S.

## 5. Conclusions 

Compared with the parent, the mutant enzyme G395S /A191T had a *K*_m_ of 0.21 mM and a catalytic efficiency *K*_cat_ /*K*_m_ of 5.38 mM^−1^∙s ^−1^, which was 4.8 times higher than that of the parent. The conditions for L-tryptophan synthesis by the mutated enzyme were an L-serine concentration of 50 mmol/L, a reaction temperature of 40 °C, a pH of 8, a reaction time of 12 h, and an L-tryptophan yield of 81%.

## Figures and Tables

**Table 1:** Primers used in this study.

Primer	Sequence (5′ - 3′)
G395S For	(5′-AACTTTCGTGCTNNNCTTTAGACT-3′)
G395S Rev	(5′-TTTTGCTGGTACANNNAAATCCTTC-3′)

**Table 2:** B-factor values of some of the amino acid residues of tryptophan synthase.

Rank	Residue Sequence Number	Amino Acid	B-Factor Value
1	395	Gly	95.24
2	394	Arg	93.55
3	382	Lys	93.34
4	393	Ala	92.39
5	392	Lys	89.52
6	383	Asp	88.7
7	313	His	88.7
8	257	Pro	87.61
9	329	Asp	86.18
10	233	Gly	86.17
11	191	Ala	86.06
12	234	Gly	85.96
13	18	Pro	85.83
14	258	Gly	85.79
15	388	His	85.68
16	17	Val	85.64
17	378	Gly	85.18
18	190	Thr	85.01
19	256	Glu	83.42
20	232	Gly	82.93

**Table 3:** Kinetic parameters of tryptophan synthase and its mutants.

Mutations	*K*_m_ (mM)	*k*_cat_ (s^−1^)	*k*_cat_/*K*_m_ (mM^−1^∙s ^−1^)	Fold Change over WT
WT	0.31 ± 0.11	0.35 ± 0.09	1.12 ± 0.07	1
G395S	0.32 ± 0.14	0.42 ± 0.11	1.31 ± 0.10	1.16
G395S/A191T	0.21 ± 0.10	1.13 ± 0.12	5.38 ± 0.11	4.80

Mean values of three independent replicate ± standard deviation.
